# α-Ketoglutaric acid: a new chance for male fertility preservation

**DOI:** 10.1093/lifemeta/loac015

**Published:** 2022-08-23

**Authors:** Fucheng Dong, Wei Li

**Affiliations:** Institute of Reproductive Health and Perinatology, Guangzhou Women and Children’s Medical Center, Guangzhou Medical University, Guangzhou 510623, China; State Key Laboratory of Stem Cell and Reproductive Biology, Institute of Zoology, Chinese Academy of Sciences, Beijing 100101, China; Institute of Reproductive Health and Perinatology, Guangzhou Women and Children’s Medical Center, Guangzhou Medical University, Guangzhou 510623, China

**In a recent study published in *Life Metabolism*, Xu *et al.* report that the α-ketoglutaric acid (AKG)/2-oxoglutarate receptor 1 (OXGR1) signaling plays an important role in the regulation of sperm maturation by maintaining epididymal fluid acid–base balance in epididymal smooth muscle cells, suggesting that AKG may provide a new chance for nutritional intervention of some male infertility.**

With the development of science and technology, the overall health conditions have been greatly improved, but the reproductive health issue is still challenging. Infertility is currently the third most common disease affecting human health, with ~50% of cases being related to male infertility [1]. Considering that the genetic background of populations cannot significantly change within half a century, the changes in environment and the lifestyle should be the major cause for most of the infertility. The effects of environmental pollution on male fertility have been intensively investigated [2], while the mechanism by which lifestyle affects male fertility is still elusive. The lifestyle may affect male fertility by altering our metabolic processes. The tricarboxylic acid cycle (TCA) is a common metabolic pathway in nearly all eukaryotes, and it has been demonstrated that extra-mitochondrial citrate synthase (eCS) is produced in the sperm head, and eCS can suppress age-associated male infertility [3]. As a TCA intermediate, AKG plays a critical role in the regulation of renal HCO_3_^−^ secretion and salt reabsorption via its receptor, OXGR1 [4]. Considering that pH value and HCO_3_^−^ concentration are also essential for the maturation of sperm in the epididymis luminal fluid, Xu *et al.* asked whether the AKG/OXGR1 signaling pathway plays any role in sperm maturation.

**Figure 1:**
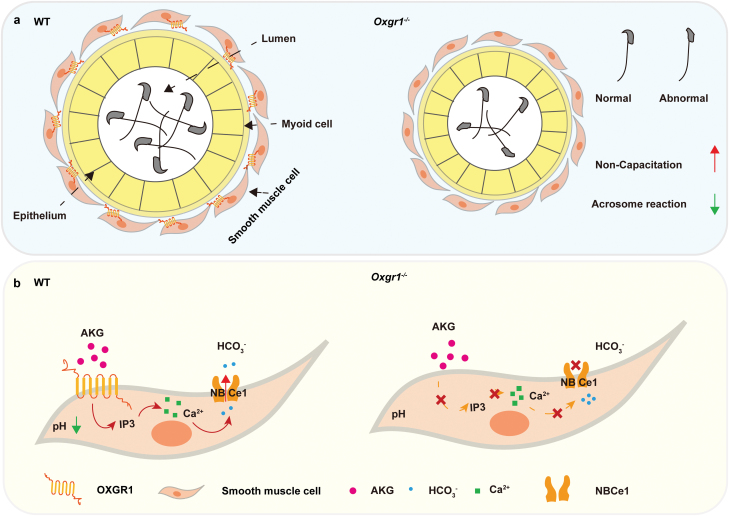
(a) Effect of *Oxgr1* knockout on sperm maturation in mice. (b) AKG promotes the release of Ca^2+^ stored in epididymal smooth muscle cells by IP3 via OXGR1, which in turn induces the expression of NBCe1 to regulate HCO_3_^−^ transport and maintain epididymal fluid acid–base balance, finally promoting sperm maturation.

The authors first examined the expression pattern of OXGR1 in reproductive tissues, and found that OXGR1 was highly expressed in the epididymis, and it colocalized with α-smooth muscle actin in the epididymal smooth muscle cells (Fig. 1a). In response to aging and heat stress, the expression of OXGR1 was significantly decreased, suggesting that it may play an important role in the epididymal smooth muscle, and its decreasing may be associated with male infertility. After generating *Oxgr1* global knockout (*Oxgr1*-GKO) mice, they found that the lumen area of the epididymis was reduced, and the fertility of the *Oxgr1*-GKO mice was decreased. Furthermore, they found that the *Oxgr1*-GKO mice had a higher rate of sperm abnormality, such as lower sperm motility and acrosome reaction (Fig. 1a). To exclude the indirect effect from other tissues, they further constructed an epididymal-specific *Oxgr1* knockdown model (*Oxgr1*-eKD) using *Oxgr1*^*Flox/Flox*^ mice and AAV-Cre-GFP virus injection. The phenotypes of these mice were similar to those of the global knockout mice. These results indicate that OXGR1 plays a direct role in epididymal sperm maturation.

In a previous study, the same research team found that exercise-induced α-ketoglutarate stimulates muscle hypertrophy and fat loss through OXGR1-dependent adrenal activation [5]. Other evidence also shows that AKG has broad therapeutic potential as an agonist of OXGR1 [6]. Therefore, the authors tested whether AKG can reverse sperm maturation disorder caused by aging and heat stress. To answer this question, they administered drinking water containing 2% AKG to mice suffering aging and heat stress for 4 weeks. They found that the proportion of abnormal sperm was significantly reduced and sperm capacitation was increased.

To further investigate whether epididymal smooth muscle OXGR1 plays a role in maintaining the epididymal luminal fluid microenvironment, the authors examined pH and HCO_3_^−^ levels in the luminal fluid of WT and *Oxgr*1-GKO epididymis. Results showed that the HCO_3_^−^ concentration and pH value were significantly reduced in the epididymis luminal fluid of *Oxgr1*-GKO mice, but not changed in the smooth muscle cells. Therefore, OXGR1 might be involved in ion transportation. Subsequently, they isolated primary epididymal smooth muscle cells from WT and *Oxgr1*-GKO mice and cultured them *in vitro*. They found that AKG promoted the release of calcium ions from smooth muscle cells through the OXGR1 receptor, which in turn affected the acid–base balance in the epididymal fluid (Fig. 1b).

Furthermore, the effect of AKG treatment on the expression of ion transporters including Na^+^/H^+^ exchanger NHE3, Na^+^/HCO_3_^−^ cotransporter NBCe1, and Cl^−^/HCO_3_^−^ exchanger AE2 [7] was tested. It was found that NBCe1 was significantly upregulated in smooth muscle cells, but not in those *Oxgr1*-GKO cells, suggesting that AKG/OXGR1 might regulate HCO_3_^−^ transport through NBCe1. Since AKG treatment could promote the release of intracellular Ca^2+^, it is possible that Ca^2+^ depletion by chelators should mimic the effect of *Oxgr1* knockout on smooth muscle cells. Indeed, they found that the Ca^2+^ chelator suppressed the expression of NBCe1 and reduced pH value following AKG treatment. This further supported the role of intracellular Ca^2+^ signaling in AKG/OXGR1-induced increase in NBCe1 expression and reduction in pH value. In addition, given that OXGR1 activates the β-isoforms of phospholipase C, which in turn promotes the release of Ca^2+^ from sarcoplasmic reticulum through the action of inositol-1,4,5-trisphosphate (IP3) [8], they tested the effect of IP3 inhibitor on AKG-induced pH reduction and NBCe1 mRNA upregulation. Results showed that IP3 inhibitor reversed the effect of AKG treatment (Fig. 1b).

In summary, this study provides convincing evidence to demonstrate that OXGR1 participates in epididymal sperm maturation. Taken together, the authors demonstrated that AKG promotes IP3-induced release of Ca^2+^ from epididymal smooth muscle cells via OXGR1, which in turn upregulates the expression of NBCe1, thereby influencing HCO_3_^−^ transport and epididymal fluid acid–base balance to promote sperm maturation (Fig. 1b). This study may provide a new way for the development of critical functional nutrients to some kinds of male infertility, and potentially link lifestyle to fertility preservation in the future.
